# Challenges associated with low rectal malignant obstruction stenting: a case report

**DOI:** 10.1093/jscr/rjad593

**Published:** 2024-09-10

**Authors:** Victor Cabrera-Bou, Eddy P Lincango, Alessandra E Cabrera, Gabriel Diaz-Pagan, Nathan Kostick, Noah Sobel, Luis F Serrano, Philip Kondylis

**Affiliations:** Surgery Department, University of Central Florida, 4000 Central Florida Blvd, Orlando, FL 32816, United States; Surgery Department, University of Central Florida, 4000 Central Florida Blvd, Orlando, FL 32816, United States; Surgery Department, University of Central Florida, 4000 Central Florida Blvd, Orlando, FL 32816, United States; Surgery Department, University of Central Florida, 4000 Central Florida Blvd, Orlando, FL 32816, United States; Surgery Department, University of Central Florida, 4000 Central Florida Blvd, Orlando, FL 32816, United States; Surgery Department, University of Central Florida, 4000 Central Florida Blvd, Orlando, FL 32816, United States; Surgery Department, University of Central Florida, 4000 Central Florida Blvd, Orlando, FL 32816, United States; Surgery Department, University of Central Florida, 4000 Central Florida Blvd, Orlando, FL 32816, United States; Colorectal Surgery, HCA Florida Osceola Hospital, 700 W Oak St, Kissimmee, FL 34741, United States

**Keywords:** anal adenocarcinoma, self-expanding metallic stents, anorectal ring

## Abstract

An ongoing debate exists regarding the feasibility of placing self-expanding metallic stents (SEMS) within 5 cm of the anal verge. Traditionally, SEMS have been considered contraindicated for patients with a malignant rectal obstruction within this region due to potential impact on the anorectal ring or anal canal, which can cause incontinence, proctalgia, and tenesmus. However, in the case of a 63-year-old female who presented with distention, abdominal pain, and diminishing stool output, the rectal exam identified a bulky fixed mass. Imaging studies revealed large bowel obstruction and high-grade stricture, with a minuscule residual lumen. Endoscopy identified a bulky mass obscuring the lumen at 5 cm from the anal verge, and biopsy confirmed adenocarcinoma. Despite the traditionally held contraindication, a 2.5 cm × 9.0 cm colonic stent was successfully deployed, leading to brisk colonic decompression. This allowed the patient to promptly undergo chemoradiotherapy.

## Introduction

Colorectal cancer presents with acute obstruction in 7–30% of cases, with higher rates if the tumor is at or distal to the splenic flexure [1]. Patients who present with acute obstruction require urgent intervention [2]. Traditionally, the solution to this is surgery with possible resection and/or ostomy formation [2]. However, this carries significant morbidity and mortality [3]. An alternative option for some patients is endoscopic stenting [4]. There is ongoing debate regarding whether self-expanding metallic stent (SEMS) placement within 5 cm of the anal verge is feasible. Endoscopic stenting acts as a bridge to definitive therapy allowing for the management of the patient prior to surgery including nutritional, oncological, and medical optimization [4]. This can increase the possibility of a successful single-stage procedure for these patients [5]. However, traditionally there is a limit on how distal these stents can be deployed [6]. SEMS has traditionally been considered contraindicated for patients with malignant rectal obstruction located within 5 cm of the anal verge due to the potential risk of damaging the anorectal ring or anal canal, which may lead to incontinence, proctalgia, and tenesmus. However, recent studies have challenged this notion, and some experts have demonstrated that endoscopic stenting can be safely and effectively used in selected patients with low rectal cancer, even those with obstruction close to the anal verge. This paper reports a successful case of endoscopic stenting as a palliative treatment option.

## Case report

A 63-year-old woman presented with abdominal distention, abdominal pain, and diminishing stool output. Her medical and surgical history was significant for chronic daily smoking, total hysterectomy with bilateral salpingoophorectomy and debulking of distal small bowel mass, following a high-grade small bowel obstruction from a pelvic mass 4 months prior this presentation. Patient denied history of colonoscopy. Patient was hemodynamically stable, abdomen was distended and tender, and rectal exam identified a bulky fixed mass. Laboratory results were unremarkable, except for electrolytes derangement. Carcinoembryonic antigen was 1.2.

**Figure 1:**
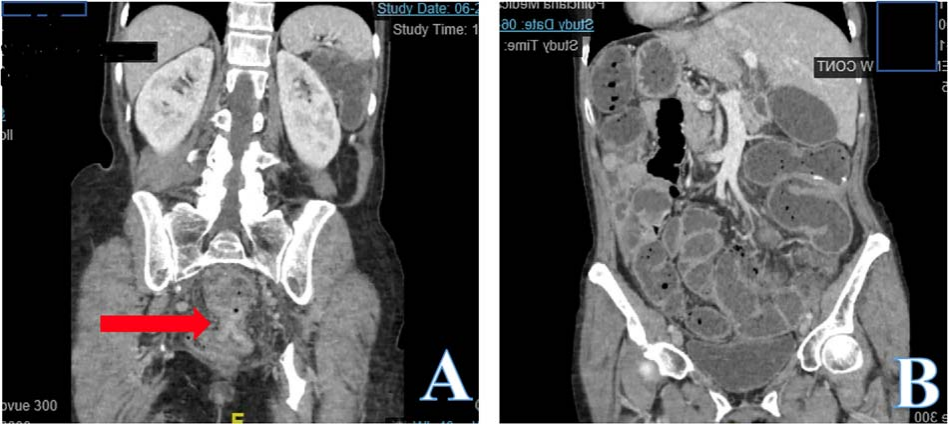
Abdomen and pelvis CT scan, coronal view (A) demonstrates low rectal concentric and symmetric near obstructing stricture indicated with the arrow. Coronal view (B) demonstrates a diffuse colonic and small bowel dilation consistent with LBO.

**Figure 2:**
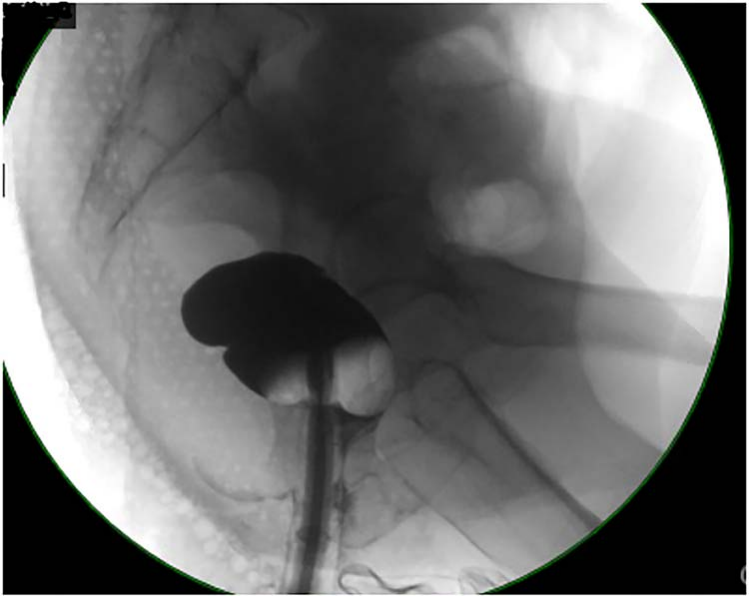
Contrast enema showing no retrograde filling due to low rectal stricture.

Computed tomography (CT) abdomen and pelvis revealed large bowel obstruction and high-grade stricture, with a miniscule residual lumen (Fig. 1). A nasogastric tube was placed for decompression, with significant output. CT chest showed no evidence of widespread disease. Gastrografin enema was performed to evaluate the obstructive lesion, and to rule out any synchronous proximal lesions, as shown in Fig. 2, no contrast passed beyond the rectal stricture.

**Figure 3:**
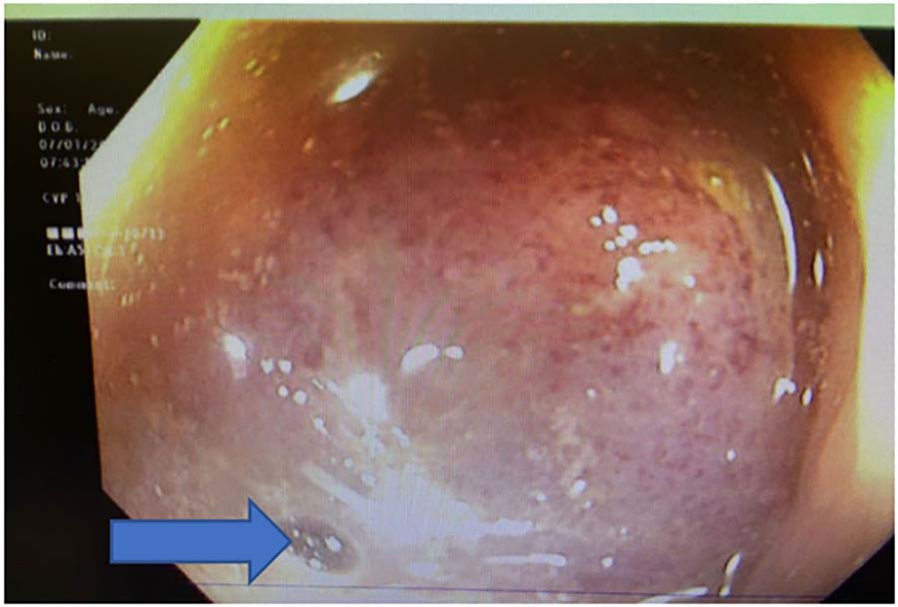
Colonoscopy showing bulky mass obscuring the lumen at 5 cm from the anal verge, blue arrow pointing at the miniscule lumen.

**Figure 4:**
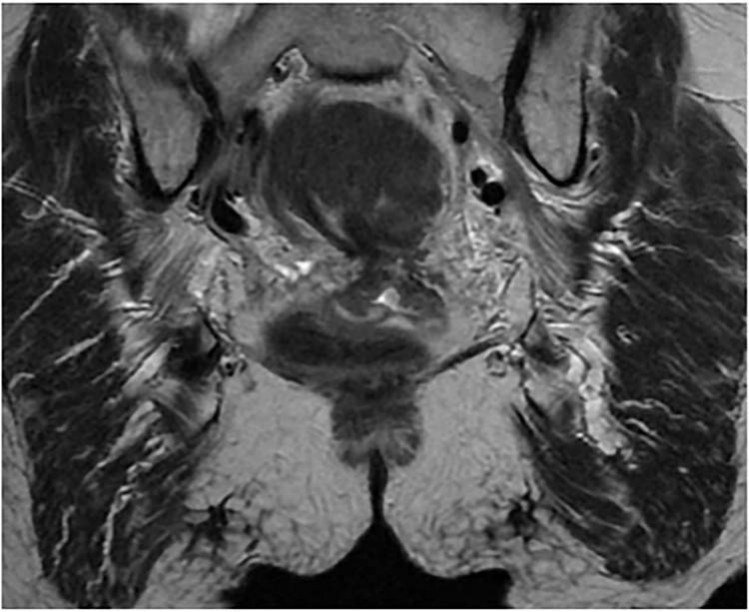
1.5 pelvis MRI, coronal view demonstrates apple core lesion at 5 cm from anal verge staged at T3N0 without involvement of anal sphincter.

On hospital Day 2, patient underwent diagnostic sigmoidoscopy. We identified a bulky mass obscuring the lumen at 5 cm from the anal verge (Fig. 3). During hospital Day 3, patient was tolerating a clear liquid diet. A 1.5 Tesla Pelvic MRI demonstrated an apple core lesion, staged at least mT3N0, sparing anal sphincters, as shown in Fig. 4. Biopsy pathology identified adenocarcinoma. During hospital Day 4, oncology was consulted and recommended neoadjuvant chemoradiation therapy as outpatient.

**Figure 5:**
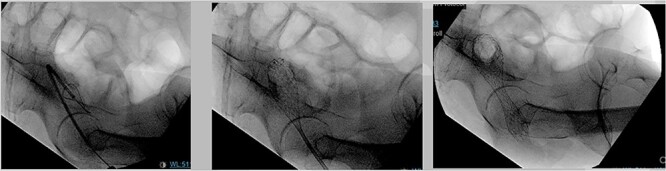
Endoscopic 2.5 cm × 9 cm. Wall flex low rectal stent placement under fluoroscopy guidance.

After extensive discussion and education with the patient in regard to surgical options, such as diverting transverse colostomy or endoscopic decompressive stent placement, the patient decided to undergo colonic stent decompression. During hospital Day 4, a 2.5 cm × 9.0 cm colonic stent was deployed, we were only able to deploy it just above the anorectal ring (Fig. 5), with brisk colonic decompression. During postoperative day (POD) 1, she had voluminous fecal incontinence for 12 hours before spontaneous resolution. Prior to discharge, a contrast enema demonstrated stent patency and excluded synchronous lesions. Patient was having liquid bowel movements and was tolerating a diet. Prior discharge a med-port was inserted for chemotherapy. Patient was successfully discharged on POD 2.

## Outpatient follow-up

The patient was followed up in the colorectal clinic 2 weeks after being discharged. On rectal examination, the stent was patent. The patient reported minimal discomfort during bowel movements and resolved incontinence. FOLFOX chemotherapy was initiated 3 weeks after stent placement, and radiation therapy for a total of 6 months was provided.

## Discussion

There are three primary indications for SEMS: obstructive colorectal cancer with the goal of bridging to surgery, unresectable obstructive malignant colorectal cancer with the goal of palliation, and benign obstructive disease. In this case, we present an example of SEMS being used in a patient with obstructive colorectal cancer as a bridge to surgery. SEMS has been shown to have technical success in up to 91% of patients and clinical success in up to 83% of patients with left-sided colorectal cancer [1]. This allows patients to be optimized for a single-stage procedure, which reduces mortality and morbidity compared to upfront surgery [2, 3]. SEMS is also associated with a shorter duration of admission, lower ICU admission rate, and quicker initiation of chemotherapy after surgery [4, 5]. Complications occur in up to 40% of SEMS with 26% requiring repeat endoscopic intervention [6]. The most common of these complications include stent migration (15%), occlusion (10%), or fistula formation (7%) [6]. One of the most feared complications is colonic perforation at the site of stent placement. Despite SEMS’s high complication rate, it is still lower than that of emergency surgery (40 vs 56%). It is frequently successful in optimizing patients so they can receive a single stage procedure [1, 2, 6]. For these reasons, SEMS is a viable alternative to emergency surgery in patients with obstructive colorectal cancers.

Despite this, SEMS has historically been avoided in low rectal lesions. Our case demonstrates that SEMS can be used in the setting of low rectal cancer. This paper describes a case with self-limited fecal incontinence with very low rectal stenting. Low rectal stenting allows for decompression of the proximal rectum and colon. Initially after deployment of the stent the increased proximal colonic pressure is distributed distally through the stent and can overwhelm the remaining anorectal segment leading to incontinence. However, as demonstrated by this case, once the proximal segment has been adequately decompressed the incontinence tends to resolve. In addition, previous literature [7, 8] has emphasized the importance of deploying stents at least 2 cm above the anal canal to avoid incontinence or tenesmus. In this patient, we deployed the stent just above the anorectal ring. Some literature [9–12] has previously described argon beam trimming of metallic stents. As our patient had an unprepped bowel, the authors elected to avoid this due to concern for combustible gas.

This case illustrates that stenting can be an effective treatment option for lesions in the low rectum. However, stenting in this location presents greater technical challenges. There is an elevated risk of transient incontinence, proctalgia, and tenesmus associated with SEMS in this position. Nevertheless, as easily removable metal stents become more commonly placed, these risks can be mitigated or reversed. This development should encourage endoscopists to consider stenting in this previously contraindicated location.

## Data Availability

Data can be made available upon request to the corresponding author.
